# Persistent hypoglycemia as an early, atypical presentation of hepatocellular carcinoma: A case report and systematic review of the literature

**DOI:** 10.3892/ol.2014.2365

**Published:** 2014-07-18

**Authors:** CHEN-YEN TSAI, SHOU-CHU CHOU, HSIEN-TA LIU, JIUNN-DIANN LIN, YING-CHIN LIN

**Affiliations:** 1 Department of Family Medicine, Shuang Ho Hospital, Taipei Medical University, New Taipei 23561, Taiwan, R.O.C.; 2 Graduate Institute of Clinical Medicine, Chang Gung University, Tao-Yuan 33302, Taiwan, R.O.C.; 3 Department of Family Medicine, School of Medicine, College of Medicine, Taipei Medical University, Taipei 11031, Taiwan, R.O.C.; 4 Department of Endocrinology and Metabolism, Shuang Ho Hospital, Taipei Medical University, New Taipei 23561, Taiwan, R.O.C.

**Keywords:** hypoglycemia, hepatocellular carcinoma, review, steroids, signs and symptoms

## Abstract

The current study presents a case of persistent hypoglycemia as the initial manifestation of advanced hepatocellular carcinoma (HCC), as well as a systematic review of the management of hypoglycemia associated with HCC. A 42-year-old female presented with loss of consciousness and a blood glucose level of 30 mg/dl (normal range, 80–140 mg/dl). Abdominal ultrasound and computed tomography were performed to investigate tenderness in the right upper quadrant, and the results revealed a hepatic mass of 15 cm in diameter, with metastasis. A diagnosis of insulinoma was ruled out by examining the insulin level. Prednisolone treatment was ineffective for relieving the persistent hypoglycemia, however, a single dose of palliative radiotherapy reduced the hypoglycemic episodes to once monthly. Due to the advanced disease, the patient refused further treatment, with the exception of a palliative therapy with glucose fluid. The patient succumbed to pneumonia with sepsis. A systematic review of the literature indicated that steroids were the most commonly used drug for hypoglycemia associated with HCC, however, in the majority of cases no effect was noted as observed in this study. Cytoreduction by surgery or systemic chemotherapy has been the most effective treatment. Although rare, hypoglycemia may be the initial symptom of HCC. Cytoreduction is the most effective method of treating hypoglycemia associated with HCC.

## Introduction

Hypoglycemia has been reported to occur in 4–27% of patients with hepatocellular carcinoma (HCC), and is associated with an unfavorable prognosis and poor survival (1). In total, ~23% of cases of non-islet cell tumor hypoglycemia (NICTH) are associated with HCC (2), typically appearing during the final stage of the disease (3). Although hypoglycemia associated with HCC is a recognized disease entity, there is currently a lack of effective treatment or prevention.

The aim of the current study was to present a case of HCC with hypoglycemia as the initial manifestation, and to perform a systematic review of the literature, with a focus on treatments other than glucose infusion and frequent feeding. Patient provided written informed consent.

## Case report

In November 2010, a 42-year-old female presented to the Emergency Department of the Shuang Ho Hospital, Taipei Medical University (Taipei, Taiwan) due to loss of consciousness. Once consciousness had been regained, the patient reported no symptoms other than malaise and poor appetite during the previous week, and denied any recent alcohol consumption. No other medical history was reported, with the exception of depression. A physical examination revealed only right upper abdominal discomfort.

In the Emergency Department, the patient’s vital signs were as follows: Body temperature, 39.2°C; blood pressure, 114/60 mm Hg; heart rate, 82 beats/min; and respiratory rate, 14 breaths/min. The patient’s Glasgow Coma Scale score was E4V1M4 (4). Laboratory testing revealed a blood glucose level of 30 mg/dl (normal range, 80–140 mg/dl), a white blood cell count of 11,800/μl (normal range, 4,800–10,800/μl; 82% neutrophils), a serum glutamic oxaloacetic transaminase level of 108 IU/l (normal range, 5–40 IU/l) and a serum glutamic pyruvic transaminase level of 74 IU/l (normal range, 5–40 IU/l). The hepatitis B surface antigen level was markedly elevated (6,216 index) and the test for the hepatitis C virus antibody was negative. Lumbar puncture and drug levels excluded drug effects and central nervous system infection. The initial diagnosis was hypoglycemia and hepatitis B infection.

**Figure 1:**
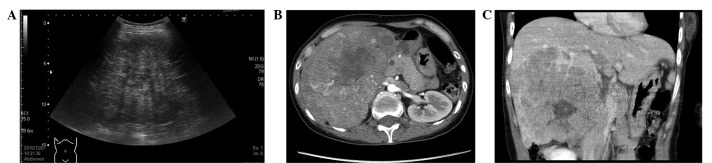
(A) Abdominal ultrasound images revealing a large (>12 cm) mixed echoic hepatic tumor, with ill-defined margins. Computed tomography scans revealing (B) a large hepatic adenoma, with multiple daughter nodules, and (C) multiple daughter nodules in the right lobe of the liver on the coronal view.

The patient was admitted, and electroencephalography and brain magnetic resonance imaging revealed no significant findings. The patient’s insulin level was <0.5 IU/ml (normal range, 3.0–25.0 IU/ml), while the blood glucose level was 74 mg/dl, thus excluding a diagnosis of insulinoma. A diagnosis of NICTH was subsequently considered. Abdominal ultrasound revealed a mixed echoic hepatic tumor with ill-defined margins (Fig. 1A), and abdominal computed tomography revealed a large mass in the liver with local metastasis (Fig. 1B and C). The serum α-fetoprotein level was elevated (469,013 ng/ml; normal range, <20 ng/ml) and the C-peptide concentration was reduced (0.06 ng/ml; normal range, 0.81–3.85 ng/ml). Therefore, a diagnosis of HCC and NICTH was formed.

Due to the advanced disease stage, the patient refused active treatment and subsequently received only palliative treatment, including prednisolone for the prevention of hypoglycemia. Following discharge, the patient presented to the Emergency Department approximately twice a month due to an altered level of consciousness. For these episodes, the patient received a 50% dextrose infusion to relieve the persistent hypoglycemia, followed by a 10% dextrose infusion to maintain glucose homeostasis. In September 2011, a single dose of palliative radiotherapy (5,000 cGy of 22 fractions) for one month reduced the hypoglycemic episodes to once monthly. The patient was hospitalized with pneumonia in January 2012. One week later this progressed to sepsis and the patient succumbed.

## Discussion

A search of the literature was conducted using Medline (www.ncbi.nlm.nih.gov/pubmed), Cochrane (www.thec-ochranelibrary.com/view/0/index.html), EMBASE (www.elsevier.com/online-tools/embase) and Google Scholar (scholar.google.com) on June 30, 2013, using combinations of the following keywords: ‘Hepatocellular carcinoma’, ‘hepatoma’, ‘hypoglycemia’ and ‘hypoglycemic’. The inclusion criteria were as follows: i) Primary HCC; ii) hypoglycemia considered as a paraneoplastic symptom, not an adverse event of anticancer treatment; and iii) study published in the English language. Studies that did not report the management of hypoglycemia, or review articles and editorials were excluded.

Studies were identified according to this search strategy, used by two independent reviewers. Where there was uncertainty with regard to eligibility, a third reviewer was consulted. The reference lists of the relevant studies were also searched. The following information and results were extracted from the studies that met the inclusion criteria: The name of the first author, year of publication, study design, number of subjects, patient age and gender, and treatment regimen for hypoglycemia other than glucose infusion, feeding or supportive care.

**Figure 2:**
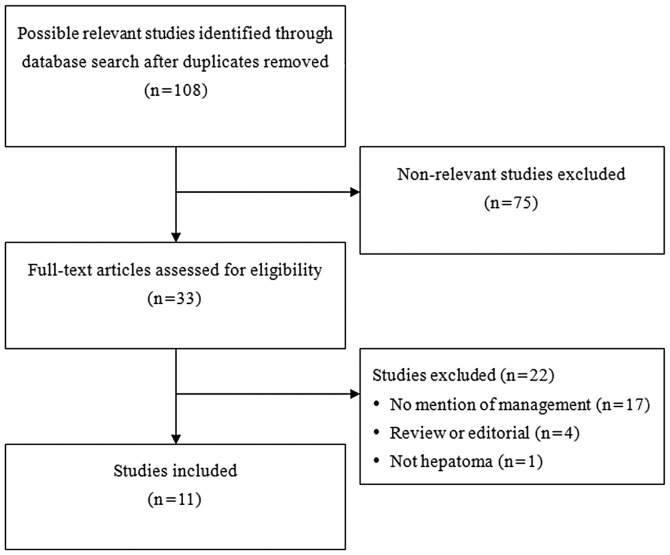
Flow diagram of study selection.

A total of 108 potentially relevant studies were identified by the initial review, and following the application of the inclusion and exclusion criteria, 11 studies remained for inclusion in the systematic review. A flow diagram of the study selection is shown in Fig. 2, and the 11 included studies (1,5–14) are summarized in Table I. Steroids were the most commonly used drug for the management of hypoglycemia; however, in the majority of cases, no effect, or only a transient effect with respect to increasing glucose levels, was noted. In one case, growth hormone was administered (8 units intramuscularly every 8 h) and although the glucose levels increased, the hypoglycemia was not resolved (8). Diazoxide was used in another case, but no beneficial effect was noted (10). Cytoreduction by surgical removal of the tumor resulted in resolution of the hypoglycemia in one case (12), while percutaneous ethanol injection of the tumor resulted in the resolution of the hypoglycemia in another case (1). Furthermore, systemic chemotherapy resulted in a transient resolution in one case (9).

Paraneoplastic manifestations of hypoglycemia commonly occur in association with large abdominal tumors; however, hypoglycemia is a rare initial presentation of HCC. Patients with hypoglycemia and HCC have low concentrations of insulin and C-peptide (15), which rules out insulin overproduction as a cause.

Two types of hypoglycemia have been associated with HCC (16). Type A hypoglycemia occurs in end-stage HCC, with rapid tumor growth, and once hypoglycemia occurs, mortality may occur within two weeks (3,17). Hypoglycemia is typically mild and is caused due to the inability of a severely damaged liver to meet the body’s demand for glucose (3,17). Type B hypoglycemia, as observed in the present case, accompanies slow-growing tumors, usually occurs between two and 10 months prior to mortality and is severe (3,17). Patients typically present with marked alterations in consciousness, convulsions or coma. Type B hypoglycemia is considered to be due to the defective processing of the precursor to insulin-like growth factor II by tumor cells. The defective precursor passes more easily across capillary membranes, resulting in increased glucose uptake (3,15,17).

Surgical excision and chemotherapy may effectively reduce the tumor volume and relieve hypoglycemia (18). However, a large tumor volume usually precludes tumor resection in type B hypoglycemia. Frequent feeding, parenteral dextrose infusion, corticosteroids and growth hormones have all been used for the management of hypoglycemia with varying degrees of success (3).

The results of the systematic review presented in the current study suggested that the most effective management for hypoglycemia resulting from HCC is cytoreduction by surgery (18), chemotherapy (13) or ethanol injection, as described by Saigal *et al* (1). In the present patient case, cytoreduction by radiotherapy was also effective in reducing the frequency of the hypoglycemic episodes. However, in the majority of cases, cytoreduction is not an option due to the terminal condition at diagnosis. No effective and convincing pharmaceutical treatments were noted from the literature review. Steroids were the most frequently used drug for the management of hypoglycemia resulting from HCC, however, the results were mixed. A long-acting steroid accompanying a midnight meal appeared to be more effective than short-acting steroids, however, the effect was transient (11).

The span of the publication of the 11 studies ranged between 1956 and 2011, and during that period of time diagnostic modalities and treatments have changed greatly. Although accurate diagnostic tools and interventions for HCC now exist, and the mechanism of the hypoglycemia resulting from HCC has been characterized, the results of the review indicate that effective management for hypoglycemia resulting from HCC is lacking.

In conclusion, NICTH must be considered when evaluating patients with persistent hypoglycemia, and although rare, hypoglycemia may present as an initial symptom of HCC. Cytoreduction is the most effective method for treating hypoglycemia associated with HCC, and while steroids are frequently used, they are not effective in the majority of cases.

## Figures and Tables

**Table I:** Summary of the studies included in the systematic review.

First author/s, year (ref.)	Age, years/gender	Treatment regimen for hypoglycemia^a^	Response to treatment
McFadzean and Yeung, 1956 (5)	-/-b	200 mg cortisone daily	Cortisone had no effect in one patient; in two patients, the glucose level increased, but withdrawal of cortisone resulted in return of hypoglycemia
Klein and Klein, 1959 (6)	62/F	200 mg cortisone daily	No beneficial effect
Schonfeld *et al,* 1961 (7)	27/M	60 mg methylprednisolone daily	No effect
Wing *et al*, 1991 (8)	30/M	8 units growth hormone, intramuscularly	At 30 min after discontinuation of the glucose infusion, the decremental every 8 h for two days change in blood glucose level was −2.8 mmol/l; at 60 min after therapy with growth hormone or prednisolone, the decremental change was attenuated to −1.2 mmol/l
Wing *et al*, 1991 (8)	27/M	1 mg/kg/day prednisolone, orally for two days	At 30 min after discontinuinuation of glucose infusion, the decremental change in blood glucose level was 3.2 mmol/l; at 60 min after therapy with growth hormone or prednisolone, the decremental change was attenuated to −1.5 mmol/l
Yonei *et al,* 1992 (9)	62/M	Glucagon, oral prednisolone or chemotherapy	Not effective or only a transient effect (Adriamycin and cisplatin)
Hof and Vassilopoulou-Sellin, 1998 (10)	34/M	Diazoxide	No beneficial effect
Saigal *et al*, 1998 (1)	24/F	Percutaneous ethanol injection (weekly) for three cycles	Hypoglycemic attacks became infrequent and the intravenous glucose requirement markedly decreased
Thipaporn *et al,* 2005 (11)	36/M	40 mg/day prednisolone, followed by 2 mg/day dexamethasone	No hypoglycemia on dexamethasone provided the patient received food at midnight; one month of follow-up
Nikeghbalian *et al,* 2006 (12)	77/M	Complete surgical removal of the tumor	Hypoglycemia resolved
Kampitak, 2008 (13)	16/M	Systemic chemotherapy with doxorubicin	Temporary resolution of hypoglycemia
Matsuyama *et al*, 2011 (14)	69/M	Dexamethasone	No beneficial effect

a All patients received glucose and feeding.

b Three patients studied; age/gender not disclosed.

F, female; M, male.
